# Plasma concentrations of extracellular matrix protein fibulin-1 are related to cardiovascular risk markers in chronic kidney disease and diabetes

**DOI:** 10.1186/1475-2840-12-6

**Published:** 2013-01-07

**Authors:** Alexandra Scholze, Else-Marie Bladbjerg, Johannes J Sidelmann, Axel CP Diederichsen, Hans Mickley, Mads Nybo, W Scott Argraves, Peter Marckmann, Lars M Rasmussen

**Affiliations:** 1 Clinical Research Unit, Department of Nephrology, Odense University Hospital, Kloevervaenget 6, Odense, 5000, Denmark; 2 Unit for Thrombosis Research, Institute of Public Health, Department of Clinical Biochemistry, Hospital of Southwest Denmark, University of Southern Denmark, Esbjerg, Denmark; 3 Department of Cardiology, Odense University Hospital, Odense, Denmark; 4 Department of Regenerative Medicine and Cell Biology, Medical University of South Carolina, Charleston, SC, USA; 5 Department of Clinical Biochemistry, Odense University Hospital, Odense, Denmark

**Keywords:** Fibulin-1, Arterial stiffness, Cardiovascular disease, Kidney disease, Diabetes

## Abstract

**Background:**

Fibulin-1 is one of a few extracellular matrix proteins present in blood in high concentrations. We aimed to define the relationship between plasma fibulin-1 levels and risk markers of cardiovascular disease.

**Methods:**

Plasma fibulin-1 was determined in subjects with chronic kidney disease (n = 32; median age 62.5, inter-quartile range 51 – 73 years) and 60 age-matched control subjects. Among kidney disease patients serological biomarkers related to cardiovascular disease (fibrinogen, interleukin 6, C-reactive protein) were measured. Arterial applanation tonometry was used to determine central hemodynamic and arterial stiffness indices.

**Results:**

We observed a positive correlation of fibulin-1 levels with age (r = 0.38; p = 0.033), glycated hemoglobin (r = 0.80; p = 0.003), creatinine (r = 0.35; p = 0.045), and fibrinogen (r = 0.39; p = 0.027). Glomerular filtration rate and fibulin-1 were inversely correlated (r = −0.57; p = 0.022). There was a positive correlation between fibulin-1 and central pulse pressure (r = 0.44; p = 0.011) and central augmentation pressure (r = 0.55; p = 0.001). In a multivariable regression model, diabetes, creatinine, fibrinogen and central augmentation pressure were independent predictors of plasma fibulin-1.

**Conclusion:**

Increased plasma fibulin-1 levels were associated with diabetes and impaired kidney function. Furthermore, fibulin-1 levels were associated with hemodynamic cardiovascular risk markers. Fibulin-1 is a candidate in the pathogenesis of cardiovascular disease observed in chronic kidney disease and diabetes.

## Background

The extracellular matrix protein fibulin-1 is emerging as a new factor in cardiovascular disease. The plasma concentration of fibulin-1 is a predictor of all cause and cardiovascular mortality in patients with diabetes mellitus [1].

Fibulin-1 belongs to a family of extracellular matrix (ECM) proteins with functional associations with elastic fibers and basement membranes. Fibulin-1 interacts with a number of ECM molecules, but whether it serves a structural role has not yet been established [2]. Fibulin-1 is expressed in embryonic and adult vascular smooth muscle cells (VSMC) [3,4]. In adult blood vessels fibulin-1 is deposited in the medial layers surrounding VSMCs and in association with elastic laminae and in the adventitial ECM [1,4].

Fibulin-1 mRNA expression is increased in a model of cardiomyopathy [5], and fibulin-1 plasma levels positively correlate with plasma N-terminal pro-B-type natriuretic peptide, a cardiac marker of pressure or volume overload [1,6].

Several studies link fibulin-1 to atherosclerosis and thrombosis, including studies showing that it binds fibrinogen, mediates platelet adhesion, is a component of newly formed fibrin-containing thrombi and that it accumulates in human atherosclerotic lesions [7-10].

Based on these findings fibulin-1 may be involved in the development or progression of cardiovascular disease. We tested the hypothesis that plasma fibulin-1 levels are associated with cardiovascular risk markers in patients with chronic kidney disease and diabetes mellitus.

## Methods

### Study subjects

Chronic kidney disease (CKD) patients were recruited from the outpatient population of the Department of Nephrology, Odense University Hospital, Odense, Denmark. Written informed consent was obtained from each patient. The protocol was in accordance with the ethical standards of the Declaration of Helsinki and was approved by the regional ethics committee (reference number: S-20090061). Fifty-seven measurements were performed in 32 patients; 16 patients with CKD stage 1 – 5 without hemodialysis therapy and 16 patients with hemodialysis therapy. In 25 of the study participants two measurements per patient were performed with an interval of 2 month. Population characteristics are described in Table 1.

**Table 1:** Population characteristics of chronic kidney disease study participants

**Characteristics**	
Age, years	62.5 (51 – 73)
Sex, men, n (%)	25 (78)
Body mass index, kg/m^2^	24.8 (21.7 – 28.1)
Smoking, n (%)	18 (56)
Underlying kidney disease, n (%)	
Diabetic nephropathy	2 (6)
Nephrosclerosis	9 (28)
Glomerulonephritis	4 (13)
Others	17 (53)
Disease prevalence, n (%)	
Diabetes	11 (34)
Hypertension	25 (78)
Peripheral artery disease	2 (6)
Coronary artery disease	9 (28)
Stroke	5 (16)
eGFR, mL/min/1.73 m^2, *^	39.9 (23.3 – 58.3)
Kt/V^†^	1.1 (0.9 – 1.4)
Medication, n (%)	
Phosphate binder	16 (50)
Erythropoietin analog	15 (47)
Platelet aggregation inhibitor	8 (25)
Diuretic	7 (22)
ACE inhibitor/AT receptor antagonist	14 (44)
Calcium antagonist	15 (47)
ß-Blocker	12 (38)

Values are medians (25% - 75% percentile) or numbers (percentages), n = 32.

Abbreviations: *ACE* angiotensin converting enzyme, *AT* angiotensin, *eGFR* estimated glomerular filtration rate, Kt/V = dialysis dose.

^*^ n = 16, eGFR determination only in patients without hemodialysis therapy.

^†^ n = 8.

CKD was defined by structural or functional abnormalities of the kidney with or without decreased glomerular filtration rate (GFR) or estimated glomerular filtration rate (eGFR) less than 60 mL/min/1.73 m^2^ for more than 3 months. Estimated GFR was calculated according to the Modification of Diet in Renal Disease formula [11].

Hemodialysis (HD) patients were routinely dialyzed for 4 to 5 hours, three times weekly. Dialyses were performed using standardized techniques with bicarbonate-based dialysates and controlled ultrafiltration rate. In HD patients, the dialysis dose was calculated as the amount of plasma cleared of urea divided by the urea distribution volume (Kt/V). Diabetes was defined using criteria of the American Diabetes Association or by a history of or current treatment for diabetes mellitus.

Control samples for fibulin-1 determination were obtained from 60 age- and sex-matched subjects without kidney disease and without diabetes from the DanRisk study population for the sole purpose of fibulin-1 concentration comparison (age, median 60 years, range 60 to 61; sex, 46 men, 77%; BMI (body mass index), median 27.1 kg/m^2^, range 24.7 to 30.7; smoking, 21 participants, 35%). Control samples were analyzed in the same time frame and with the same pre-analytical conditions as the study samples. The DanRisk study population has been described previously [12].

### Clinical and Biochemical Assessment

Examinations and blood samplings were performed between 7 and 10 am. Examinations and blood sampling in HD patients were performed before HD sessions. Analyses were performed at the laboratories of the participating departments according to current laboratory standards.

Plasma samples were obtained from EDTA-anticoagulated blood and stored at −80°C. An ELISA for the determination of fibulin-1 concentration was performed as described by our group previously [1]. Briefly, 96-well plates were coated with rabbit anti human fibulin-1 IgG (Rb2954 IgG; Argraves et al., Cell, 1989) and blocked with bovine serum albumin [13]. Plasma samples were used at a 1:1000 dilution and, after binding to the immobilized antibodies, were incubated with anti-human fibulin-1 IgG (mouse monoclonal, 3A11; Array Genetics, Newtown, Connecticut, USA). Europium-labeled rabbit anti-mouse IgG (AD0124, Perkin Elmer, Skovlunde, Denmark) was used as secondary antibody quantified by time-resolved fluorescence on DELFIA (Perkin Elmer). Final fibulin-1 concentrations were derived from a standard curve. The inter-assay variation was ≤ 10%.

Plasma samples for C-reactive protein (CRP), fibrinogen, and interleukin-6 (IL6) were stored at −80°C. Fibrinogen and CRP were determined with a nephelometric method (Siemens Healthcare Diagnostics Inc., Marburg, Germany). A commercial ELISA was used to measure concentrations of IL6 (Quantikine HS, R&D Systems, Abingdon, UK). The inter-assay coefficients of variation were: < 10% for fibrinogen, < 6% for CRP, < 7% for IL6.

Glycated hemoglobin (HbA_1C_) was determined using high-performance liquid chromatography as fraction of total hemoglobin A0. Analyses were performed on a Tosoh G7 automatic analyzer (Medinor, Broendby, Denmark).

Blood pressure (BP) was measured after at least 10 minutes of supine resting in a quiet room prior to blood sampling. The measurements were performed with an automated device (Omron M6, Omron HealthCare Europe B.V., Hoofddorp, Netherlands).

For pulse wave analysis an applanation tonometer (Millar, SPT-301B, Houston, Texas, USA) was applied to the radial artery. The recorded radial pressure waveforms and a corresponding brachial BP can be used to generate the ascending aortic pressure waveform. For this purpose a validated transfer function is used by the SphygmoCor^®^ system (version 7.0, Atcor Medical, Sydney, Australia) [14]. We analyzed systolic aortic BP (SBP_aortic_), diastolic aortic BP (DBP_aortic_), and aortic pulse pressure (PP_aortic_).

The effect of pulse wave reflection on the incident central pulse wave was evaluated using central augmentation pressure (CAP), which was determined from the difference between the first and the second aortic systolic pressure wave peaks. Aortic augmentation index (AIx_aortic_) was derived from CAP expressed as percentage of the central pulse pressure. Since the AIx depends on heart rate we also analyzed the AIx normalized to a heart rate of 75 beats per minute (AIx@75_aortic_). Aortic pulse wave velocity (PWV_aortic_) was assessed as a direct measure of arterial stiffness and determined between a carotid artery and femoral artery recording site [15]. The distance between the two measurement sites was determined by the subtraction method, hence by subtracting the distance between the sternal notch to the carotid artery from the sternal notch to the femoral artery. Patients rested for 10 minutes in a supine position before the measurements.

### Statistics

In those patients were the measurements were performed twice the mean of the obtained values was used for the analyses. Normal distribution of continuous variables was tested by D’Agostino & Pearson omnibus normality test. Fibrinogen, IL6, CRP, creatinine, and PWV did not show a normal distribution. Non-parametric tests were therefore applied where necessary. Continuous data are reported as median (25% - 75% percentile). Categorical variables are reported as numbers and percentages. Non-parametric bivariate correlation analysis (Spearman) was performed. Multiple linear regression analysis with backward selection was used to judge variables of significance for the prediction of plasma fibulin-1 concentration.

Analyses were performed with GraphPad prism software (version 5.0, GraphPad Software, San Diego, CA, USA) and SPSS software (release 17.0, SPSS Inc., Chicago, IL, USA). All statistical tests were two-sided and p-values less than 0.05 were considered to indicate statistical significance.

## Results

The fibulin-1 study group contained 32 patients: 3 patients with CKD 2 (eGFR more than 60 ml/min/1.73 m^2^), 7 patients with CKD stage 3 (eGFR 30–59 ml/min/1.73 m^2^), 5 patients with CKD stage 4 (eGFR 15–29 ml/min/1.73 m^2^), 1 patient with CKD stage 5 without hemodialysis therapy (eGFR less than 15 ml/min/1.73 m^2^). The remaining 16 patients were on chronic hemodialysis treatment; their median dialysis vintage was 42 (13–89) months. The clinical and biochemical variables are given in Table 2.

**Table 2:** Biochemical, hemodynamic and vascular variables of chronic kidney disease study participants

**Variable**	
P-Fibulin-1, μg/mL	73.9 (54.9 – 85.3)
HbA_1C_^*^, %	7.2 (5.6 – 8.2)
P-Fibrinogen, μmol/L	11.7 (10.0 – 13.4)
P-IL6, pg/mL	5.03 (3.50 – 8.36)
P-CRP, mg/L	4.30 (1.52 – 11.86)
S-Albumin, g/l	41 (39 – 44)
P-Creatinine, μmol/L	380 (171 – 701)
P-Urea, mmol/L	15.2 (11.7 – 19.4)
SBP _brachial_, mmHg	138 (130 – 152)
DBP _brachial_, mmHg	77 (70 – 86)
SBP _aortic_, mmHg	128 (115 – 140)
DBP _aortic_, mmHg	79 (71 – 87)
PP_aortic_, mmHg	49 (34 – 59)
Heart rate, beats/min	70 (63 – 82)
CAP, mmHg	13 (8 – 21)
AIx_aortic_, %	31 (23 – 35)
AIx @75_aortic_, %	26 (21 – 32)
PWV_aortic_, m/s	10.1 (7.9 – 13.3)

Values are medians (25% - 75% percentile), n = 32.

Abbreviations: *HbA*_*1C*_ glycated hemoglobin, *IL6* interleukin 6, *CRP* C-reactive protein, *SBP* systolic blood pressure, *DBP* diastolic blood pressure, *PP* pulse pressure, *CAP* central augmentation pressure, *AIx* augmentation index, *AIx@75* augmentation index at a heart rate of 75 beats/min, *PWV* pulse wave velocity.

^*^ n = 12.

**Figure 1:**
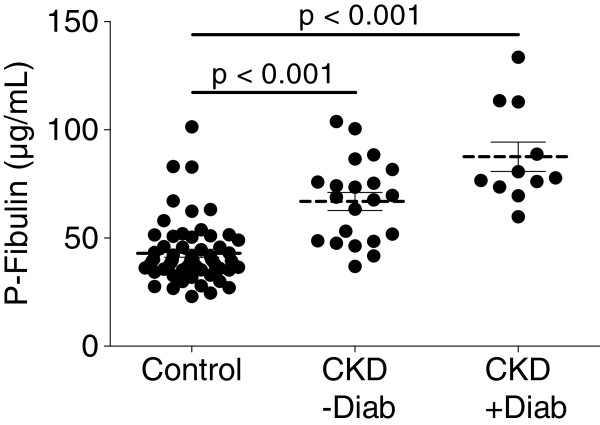
**Distribution of plasma fibulin-1 concentrations in relation to diabetes occurrence and kidney function.** Distribution of plasma fibulin-1 concentrations in age- and sex-matched control subjects without chronic kidney disease and without diabetes (Control, n = 60) and patients with chronic kidney disease (CKD) with (+Diab, n = 11) or without diabetes mellitus (−Diab, n = 21). The plasma fibulin-1 values of the groups showed a significantly different distribution (Kruskal-Wallis test p < 0.001). The differences obtained by Dunn’s multiple comparison test are indicated in the figure. Scholze et al., Figure 1.

Figure 1 shows the distribution of plasma fibulin-1 concentrations in patients with chronic kidney disease in the absence and presence of diabetes mellitus as compared to age- and sex-matched control subjects without kidney disease and without diabetes (age, median 60 years, range 60 to 61; sex, 46 men, 77%; BMI, median 27.1 kg/m^2^, range 24.7 to 30.7; smoking, 21 participants, 35%). Compared to control subjects, patients with chronic kidney disease showed higher fibulin-1 levels (p < 0.001). Furthermore, we observed significantly increased fibulin-1 concentrations in patients with chronic kidney disease plus diabetes mellitus (median, 78 μg/mL; 74 to 113 μg/mL) compared to patients with chronic kidney disease without diabetes mellitus (median, 69 μg/mL; 49 to 79 μg/mL; p < 0.050).

No differences in plasma fibulin-1 concentrations were observed between males and females (Mann–Whitney test; p = 0.964) or smokers and non-smokers (Mann–Whitney test; p = 0.444).

Next we analyzed bivariate correlations between plasma fibulin-1 concentration and clinical and biochemical variables (Table 3). We observed a positive correlation of fibulin-1 with age and HbA_1C_ concentration. Fibulin-1 levels correlated with markers of kidney function and uremia (P-creatinine, P-urea, and eGFR) with fibulin-1 levels increasing with deteriorating kidney function/increasing uremia. In hemodialysis patients, fibulin-1 concentrations were not correlated with dialysis dose (Kt/V, r = −0.43; p = 0.300) or dialysis vintage (r = 0.17; p = 0.537).

**Table 3:** Bivariate correlation analysis of plasma fibulin-1 in chronic kidney disease study participants in relation to clinical and biochemical variables (n = 32)

**Variable**	**r**	**p**
Age	0.38	0.033
HbA_1C_*	0.80	0.003
P-Fibrinogen	0.39	0.027
P-IL6	0.25	0.174
P-CRP	0.24	0.195
S-Albumin	−0.30	0.102
P-Creatinine	0.35	0.049
P-Urea	0.51	0.004
eGFR^†^	−0.57	0.022
SBP_aortic_	0.19	0.302
DBP_aortic_	−0.41	0.019
PP_aortic_	0.44	0.011
Heart rate	−0.35	0.047
CAP	0.55	0.001
AIx_aortic_	0.47	0.007
AIx@75_aortic_	0.23	0.203
PWV_aortic_	0.26	0.166

Abbreviations: *HbA*_*1C*_ glycated hemoglobin, *IL6* interleukin 6, *CRP* C-reactive protein, *eGFR* estimated glomerular filtration rate, *SBP* systolic blood pressure, *DBP* diastolic blood pressure, *PP* pulse pressure, *CAP* central augmentation pressure, *AIx* augmentation index, *AIx@75* augmentation index at a heart rate of 75 beats/min, *PWV* pulse wave velocity.

^*^ n = 12,

^†^ n = 16, eGFR determination only in patients without hemodialysis therapy.

As indicated in Table 3 we observed a positive correlation between plasma levels of fibrinogen, a marker of coagulation and systemic inflammation, and plasma fibulin-1, whereas CRP and IL6 levels were not significantly associated.

We also evaluated the degree to which plasma fibulin-1 levels were associated with hemodynamic variables and established markers of arterial stiffness. Several key variables of central hemodynamics showed significant associations with plasma fibulin-1 levels (Table 3). A positive correlation was found with aortic pulse pressure (PP_aortic_) and central augmentation pressure (CAP), while a negative correlation existed with heart rate. Markers of arterial stiffness were also investigated. No association was observed between plasma fibulin-1 and central pulse wave velocity. Since the extent of pulse wave augmentation is also heart rate dependent we used aortic augmentation index and heart rate normalized AIx (AIx @75_aortic_). We did not observe a significant association of plasma fibulin-1 concentrations with AIx@75_aortic_.

Multivariable regression analysis was performed to assess the independent contributions of different variables to plasma fibulin-1 concentration. According to the results of the bivariate correlation analysis age, presence of diabetes, creatinine, and fibrinogen as a marker of inflammation and coagulation was taken into account. To avoid multicollinearity between the hemodynamic variables, CAP was chosen for the analyses. The results are presented in Table 4. The presence of diabetes, creatinine, CAP, and plasma fibrinogen concentration together explained 59% of the variability of plasma fibulin-1 concentration in patients with CKD.

**Table 4:** Multivariable regression analysis on plasma fibulin-1

	**Independent variable**	**Adjusted r**^**2**^	**F**	**β**	**p**
model		0.59	11.98		<0.001
	Diabetes			0.40	0.005
	Creatinine			0.43	0.002
	CAP			0.25	0.073
	Fibrinogen_log_			0.32	0.011

Age, presence of diabetes, creatinine, central augmentation pressure, and logarithmically transformed plasma fibrinogen concentration were used in a stepwise backward selection process (n = 32).

Abbreviations: *CAP* central augmentation pressure.

## Discussion

In the present study we show a positive association between plasma fibulin-1 concentration and several cardiovascular risk markers in patients with CKD. Our findings are in accordance with findings from earlier experimental and clinical studies which suggested links between fibulin-1, cardiovascular disease and diabetes [7,9,16,17].

In the present study, plasma fibulin-1 concentration was found to increase with age in the study population. This is in agreement with our data previously published [1]. A multivariable analysis of the current data showed that the importance of age is not retained in the simultaneous context of kidney function, presence of diabetes, CAP and fibrinogen status.

Correlations were also observed between markers of deteriorating kidney function and increasing plasma fibulin-1 concentrations, with creatinine being an independent predictor of plasma fibulin-1 concentration. These findings are in accordance with findings from a multiplex proteomic study showing that plasma fibulin-1 could be a marker of renal impairment [18].

We also found an independent association between fibulin-1 and fibrinogen, a positive acute phase protein whose blood levels are increased in CKD. Fibrin clot characteristics in CKD differ from non-CKD patients in close correlation to inflammatory level rather than to the extent of uremia [19]. Not only has fibulin-1 been shown to bind to fibrinogen and incorporate into fibrin thrombi, but fibulin-1 also qualitatively influences the polymerization of fibrin [7,9,10]. We regard these findings as important in the context of increased plasma fibulin-1 concentrations that we report here.

Our previous studies demonstrated elevated plasma concentrations of fibulin-1 in diabetic patients [1]. We confirmed these results and again saw a correlation between higher plasma fibulin-1 concentrations and higher HbA_1C_ values. The presence of diabetes was an independent predictor of elevated plasma fibulin-1 levels.

The mechanism underlying the increase of plasma fibulin-1 is not known. In patients with diabetes we showed that it exists also in those patients without microalbuminuria hence without signs of initial kidney damage [1]. It should be noted, that diabetes and CKD share important features at the molecular level, e.g. an increase in oxidative stress and advanced protein glycation [20,21]. One or several factors from this group might be involved in the regulation of plasma fibulin-1 concentration. This should be the subject of future experimental work.

An important dimension of the present study was the analysis of central hemodynamics and vascular stiffness in relation to plasma fibulin-1 concentrations. We found a correlation with aortic AIx and central augmentation pressure (CAP). CAP was retained as independent variable in the multivariable analysis. When we performed analyses of arterial stiffness markers we did not find a correlation of plasma fibulin-1 concentrations to aortic PWV and heart rate normalized aortic AIx. Thus, plasma fibulin-1 seems to be unrelated to arterial changes that result in changes of aortic PWV. Also, increased arterial stiffness that results in an increased heart rate normalized aortic AIx [22] was unrelated to plasma fibulin-1. By contrast, a relationship was found to exist between plasma fibulin-1 and the actual central augmentation the left ventricle faces in the late systole. The pressure effort of the ventricle to overcome this augmented pressure was termed “wasted” since it does not contribute to blood flow production [23].

Several potential new biomarkers for cardiovascular diseases in diabetes have appeared during recent years. These biomarkers are associated with dysfunctions in different disease pathways and consequently display both, similarities and discrepancies in their clinical appearance.

While it was shown that plasma fibulin-1 was able to predict all-cause mortality in patients with diabetes [1] the underlying mechanisms which regulate the protein amount in certain disease entities are still uncertain. A similar dilemma applies for another interesting protein that has extensively been studied in cardiovascular disease – osteoprotegerin. Plasma osteoprotegerin concentrations are positively correlated to the presence of coronary, carotid and peripheral artery disease in diabetic patients [24,25]. Elevated plasma osteoprotegerin concentrations also predicted all-cause mortality in diabetes [26]. Several interesting mechanisms for the involvement of osteoprotegerin in cardiovascular morbidity, like an increased expression of adhesion molecules by endothelial cells or a regulative effect on vascular calcification, were described (for review see [27]). Although the function of fibulin-1 in the cardiovascular system is not well defined it could be speculated that it is different from osteoprotegerin; more related to changes in the structure of the extracellular matrix. Such functional differences between biomarkers are likely to give rise to a different clinical meaning. Nevertheless, the proof of a cause-effect relation between cardiovascular disease in humans and osteoprotegerin or fibulin-1 is still lacking and the role of both proteins for diagnosis or assessment of progression of cardiovascular disease awaits further study.

The exact role of fibulin-1 in cardiovascular disease may be complex since it is associated with pathologies in both, arterial and heart tissue. In diabetes an accumulation of fibulin-1 in the arterial wall and in plasma has been described. Plasma fibulin-1 correlated significantly with left atrial volume index and plasma N-terminal pro-B-type natriuretic peptide [1]. On the other hand, a down-regulation of fibulin-1 has been described in atrial fibrillation [28].

With respect to the above described results from our study and from previously presented investigations we find it reasonable to speculate that in clinical conditions with increased plasma fibulin-1 such as diabetes or chronic kidney disease plasma fibulin-1 may be involved in disease pathways which contribute to thrombotic and cardiovascular complications. Furthermore, plasma fibulin-1 displays a potential as cardiovascular biomarker which awaits further studies.

## Conclusions

In summary, we demonstrate an increase in plasma fibulin-1 concentration in association with diabetes and impaired kidney function. A positive association exists between plasma fibulin-1 and fibrinogen levels. Also, an increase of central augmentation pressure and augmentation index resulting in higher left ventricular systolic loading is associated with higher fibulin-1 plasma concentrations. These associations of plasma fibulin-1 are compatible with the idea that fibulin-1 may have a role in the complex pathogenesis of cardiovascular disease.

## Abbreviations

ACE: Angiotensin converting enzyme; AIx: Augmentation index; AIx@75: AIx normalized to a heart rate of 75 beats per minute; AT: Angiotensin; BMI: Body mass index; BP: Blood pressure; CAP: Central augmentation pressure; CKD: Chronic kidney disease; CRP: C-reactive protein; DBP: Diastolic blood pressure; ECM: Extracellular matrix; GFR/eGFR: Glomerular filtration rate/estimated glomerular filtration rate; HbA_1C_: Glycated hemoglobin; HD: Hemodialysis; IL6: Interleukin-6; Kt/V: Dialysis dose; PP: Pulse pressure; SBP: Systolic blood pressure; VSMC: Vascular smooth muscle cells.

## Competing interests

The authors declare that they have no competing interests.

## Authors’ contributions

PM and LMR designed research; AS, PM, EMB, JJS, MN, ACPD, HM, WSA, and LMR conducted research; AS, PM, JJS, MN, ACPD, HM, WSA, and LMR wrote the paper; AS had primary responsibility for final content. All authors read, critically revised and approved the final manuscript.
